# The distance function effect on k-nearest neighbor classification for medical datasets

**DOI:** 10.1186/s40064-016-2941-7

**Published:** 2016-08-09

**Authors:** Li-Yu Hu, Min-Wei Huang, Shih-Wen Ke, Chih-Fong Tsai

**Affiliations:** 1 Department of Psychiatry, Kaohsiung Veterans General Hospital, Kaohsiung, Taiwan; 2 Department of Psychiatry, Chiayi Branch, Taichung Veterans General Hospital, Chiayi, Taiwan; 3 Department of Information and Computer Engineering, Chung Yuan Christian University, Taoyuan, Taiwan; 4 Department of Information Management, National Central University, Taoyuan, Taiwan

**Keywords:** Pattern classification, *k*-Nearest neighbor, Euclidean distance, Distance function, Medical datasets

## Abstract

**Introduction:**

K-nearest neighbor (k-NN) classification is conventional non-parametric classifier, which has been used as the baseline classifier in many pattern classification problems. It is based on measuring the distances between the test data and each of the training data to decide the final classification output.

**Case description:**

Since the Euclidean distance function is the most widely used distance metric in k-NN, no study examines the classification performance of k-NN by different distance functions, especially for various medical domain problems. Therefore, the aim of this paper is to investigate whether the distance function can affect the k-NN performance over different medical datasets. Our experiments are based on three different types of medical datasets containing categorical, numerical, and mixed types of data and four different distance functions including Euclidean, cosine, Chi square, and Minkowsky are used during k-NN classification individually.

**Discussion and evaluation:**

The experimental results show that using the Chi square distance function is the best choice for the three different types of datasets. However, using the cosine and Euclidean (and Minkowsky) distance function perform the worst over the mixed type of datasets.

**Conclusions:**

In this paper, we demonstrate that the chosen distance function can affect the classification accuracy of the k-NN classifier. For the medical domain datasets including the categorical, numerical, and mixed types of data, K-NN based on the Chi square distance function performs the best.

## Background

In pattern classification, its goal is to allocate an object represented by a number of measurements (i.e. feature vectors) into one of a finite set of classes. The *k*-nearest neighbor (*k*-NN) algorithm is one of the most widely used classification algorithms since it is simple and easy to implement. Moreover, it is usually used as the baseline classifier in many domain problems (Jain et al. 2000).

The *k*-NN algorithm is a non-parametric method, which is usually used for classification and regression problems. It is a type of lazy learning algorithms that off-line training is not needed. During the classification stage for a given testing example, the *k*-NN algorithm directly searches through all the training examples by calculating the distances between the testing example and all of the training data in order to identify its nearest neighbors and produce the classification output (Mitchell 1997).

Particularly, the distance between two data points is decided by a similarity measure (or distance function) where the Euclidean distance is the most widely used distance function. In literature, there are several other types of distance functions, such as cosine similarity measure (Manning et al. 2008), Minkowsky (Batchelor 1978), correlation, and Chi square (Michalski et al. 1981). However, there is no a comparative study of examining the distance function effect on the performance of *k*-NN.

Moreover, since the real world datasets of medical domain problems can contain categorical (i.e. discrete), numerical (i.e. continuous), or both types of data, we believe that different distance functions should perform differently over different types of datasets. This is very important for relevant decision makers to identify the ‘best’ *k*-NN classifier for medical related problems. Therefore, the aim of this paper is to provide some guidelines about which distance function used in *k*-NN is the better choice for what type of medical datasets?

The rest of this paper is organized as follows. “Literature review” section defines the pattern classification problems, overviews the idea of *k*-NN classification, and briefly describes the five well known distance functions used in this paper. “Experiments” section presents the experimental setup and results. Finally, “Conclusion” section concludes this paper.

## Literature review

### Pattern classification

The goal of pattern classification is to allocate an object represented by a number of measurements (i.e. feature vectors) into one of a finite set of classes. Supervised learning can be thought as learning by examples or learning with a teacher. The teacher has knowledge of the environment which is represented by a set of input–output examples. In order to classify unknown patterns, a certain number of training samples are available for each class, and they are used to train the classifier (Mitchell 1997).

The problem of supervised pattern recognition can be stated as follows. Given a training dataset where each training example is composed of a number of input feature variables and their corresponding class labels. An unknown function is learned over the training dataset to approximate the mapping between the input–output examples, which is able to correctly classify as many of the training data as possible.

### k-Nearest neighbor classification

**Fig. 1:**
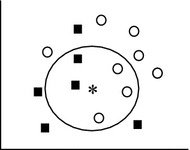
*k*-Nearest neighbor classification

The *k*-nearest neighbour (*k*-NN) classifier is a conventional non-parametric classifier (Cover and Hart 1967). To classify an unknown instance represented by some feature vectors as a point in the feature space, the *k*-NN classifier calculates the distances between the point and points in the training data set. Usually, the Euclidean distance is used as the distance metric. Then, it assigns the point to the class among its *k* nearest neighbours (where *k* is an integer). Figure 1 illustrates this concept where * represents the point. If *k* = 1, the point belongs to the dark square class; if *k* = 5, the small circle class which are the majority class of the five nearest points.

As *k*-NN does not require the off-line training stage, it main computation is the on-line ‘searching’ for the *k* nearest neighbours of a given testing example. Although using different *k* values are likely to produce different classification results, 1-NN is usually used as a benchmark for the other classifiers since it can provide reasonable classification performances in many pattern classification problems (Jain et al. 2000).

### Distance functions

To measure the distance between points *A* and *B* in a feature space, various distance functions have been used in the literature, in which the Euclidean distance function is the most widely used one. Let *A* and *B* are represented by feature vectors $A = (x_{1} ,x_{2} , \ldots ,x_{m} )$ and $B = (y_{1} ,y_{2} , \ldots ,y_{m} ),$ where *m* is the dimensionality of the feature space. To calculate the distance between *A* and *B*, the normalized Euclidean metric is generally used by

1 $$dist(A,B) = \sqrt {\frac{{\sum\nolimits_{i = 1}^{m} {(x_{i} - y_{i} )^{2} } }}{m}}$$

On the other hand, cosine similarity measure is typically used to calculate similarity values between documents in text retrieval (Manning et al. 2008) by

2 $$sim(A,B) = \frac{{\overrightarrow {A} \cdot \overrightarrow {B} }}{{\left| {\overrightarrow {A} } \right|\left| {\overrightarrow {B} } \right|}}$$

where the numerator represents the dot product of the vectors $\overrightarrow {A}$ and $\overrightarrow {B} ,$ while the denominator is the product of their Euclidean lengths.

Some other distance functions are also available for *k*-NN classification, such as Minkowsky^1^ (Batchelor 1978), correlation, and Chi square (Michalski et al. 1981).

3 $$dist\_Minkowsky(A,B) = \left( {\sum\limits_{i = 1}^{m} {\left| {x_{i} - y_{i} } \right|^{r} } } \right)^{1/r}$$

4 $$dist\_correlation(A,B) = \frac{{\sum\nolimits_{i = 1}^{m} {(x_{i} - \mu_{i} )(y_{i} - \mu_{i} )} }}{{\sqrt {\sum\nolimits_{i = 1}^{m} {(x_{i} - \mu_{i} )^{2} \sum\nolimits_{i = 1}^{m} {(y_{i} - \mu_{i} )^{2} } } } }}$$

5 $$dist\_Chi{\hbox{-}}square(A,B) = \sum\limits_{i = 1}^{m} {\frac{1}{{sum_{i}}}\left({\frac{{x_{i}}}{{size_{Q}}} - \frac{{y_{i}}}{{size_{I}}}} \right)^{2}}$$

## Experiments

### Experimental setup

Three different attribute types of datasets are chosen from the UCI machine learning repository.^2^ They are categorical, numerical, and mixed attribute types of data, which contain 10, 17, and 10 datasets respectively. Moreover, each type of datasets contains different numbers of attributes, samples, and classes in order to figure out the effect of using different types of datasets with different missing rates on the final classification accuracy.

Particularly, in the categorical attribute type of datasets, the number of attributes, samples, and classes range from 4 to 857, 106 to 12,960, and 2 to 11 respectively. For the numerical attribute type of datasets, they range from 4 to 64, 150 to 45,211, and 2 to 10 respectively. On the other hand, for the mixed attribute type of datasets the number of attributes, samples, and classes range from 6 to 20, 101 to 30,161, and 2 to 29 respectively. The detailed information of these datasets is shown in Table 1.

**Table 1:** Dataset information

Dataset	No. of instances	No. of attributes	No. of classes
Categorical datasets			
Lymphograph	148	18	4
Nursery	12,960	8	11
Promoters	106	58	2
SPECT	267	22	2
Numerical datasets			
Blood	748	5	2
Breast cancer	286	9	2
Ecoli	336	8	8
Pima	768	8	2
Mixed datasets			
Acute	120	6	2
Contraceptive	1473	9	3
Liver_disorders	345	7	2
Statlog	270	13	2

On the other hand, for *k*-NN classifier design, the *k* values are set from 1 to 15 for comparison. In addition, tenfold cross validation is used to divide each dataset into 90 % training and 10 % testing sets to train and test the *k*-NN classifier respectively. Specifically, four different distance functions, which are Euclidean distance, cosine similarity measure, Minkowsky, correlation, and Chi square, are used in the *k*-NN classifier respectively.

### Experimental results

#### Results on categorical datasets

**Fig. 2:**
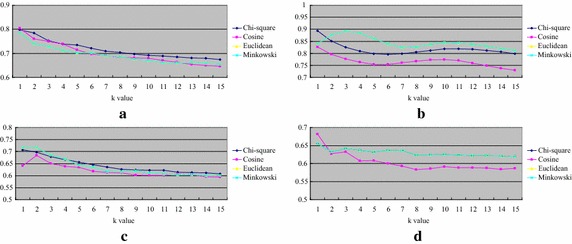
Classification accuracy of *k*-NN over categorical datasets. **a** Lymphograph, **b** nursery, **c** promoters and **d** SPECT

Figure 2 shows the classification accuracy of *k*-NN over categorical datasets. For the distance function, there is no exact winner for all of the datasets. However, overall speaking, using the Euclidean distance function is not the best metric for *k*-NN for two out of the four datasets, except for Nursery and SPECT. The classification accuracy by Euclidean and Minkowsky distance functions are almost the same, which means that using 1 or 2 for *r* does not affect the *k*-NN performance (c.f. Eq. 3). On the other hand, *k*-NN by the Chi square distance function performs best over the Lymphograph and Promoters datasets.

**Fig. 3:**
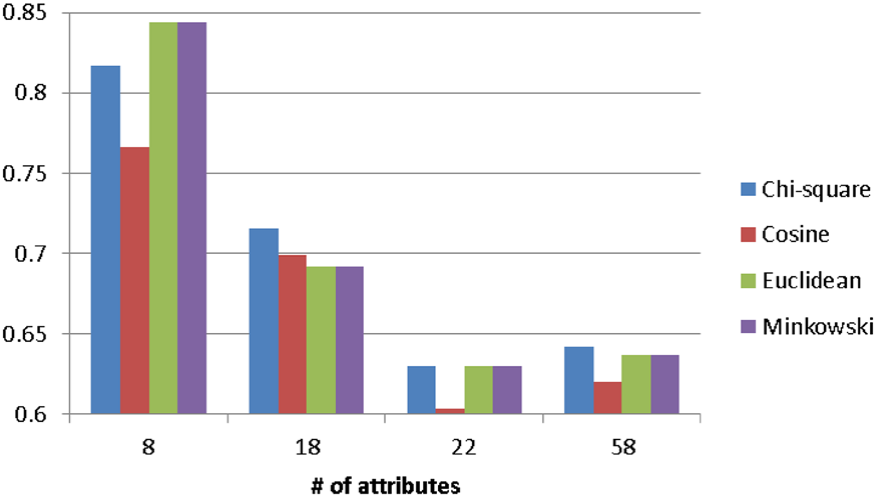
The average classification of *k*-NN over the attribute numbers of the four categorical datasets

Figure 3 shows the average classification accuracy of *k*-NN over the attribute numbers of the four categorical datasets. As we can see that when the number of attributes increases, using the Chi square distance function can make the *k*-NN classifier performs similar or slightly better than the other functions.

#### Results on numerical datasets

**Fig. 4:**
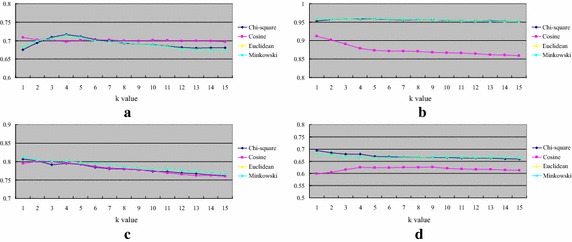
Classification accuracy of *k*-NN over numerical datasets. **a** Blood, **b** breast cancer, **c** Ecoli and **d** Pima

Figure 4 shows the classification accuracy of *k*-NN over numerical datasets. The results are opposed to the ones over the categorical datasets that *k*-NN by the Euclidean (and Minkowsky) distance function performs the best over most of the datasets, which are breast cancer, Ecoli, and Pima. On the other hand, *k*-NN by the cosine distance function only performs better than the others over the blood dataset.

**Fig. 5:**
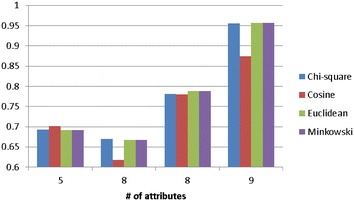
The average classification of *k*-NN over the attribute numbers of the four numerical datasets

Figure 5 shows the average classification accuracy of *k*-NN over the attribute numbers of the four numerical datasets. For most cases or larger numbers of attributes, the Euclidean (and Minkowski) and Chi square distance functions allow the *k*-NN classifier to perform very similar and better than the one using the cosine distance function.

#### Results on mixed types of datasets

**Fig. 6:**
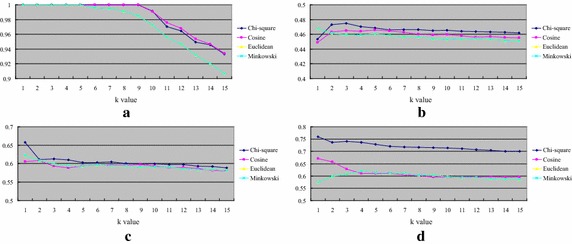
Classification accuracy of *k*-NN over mixed types of datasets. **a** Acute, **b** contraceptive, **c** Liver_disorders and **d** Statlog

Figure 6 shows the classification accuracy of *k*-NN over the mixed types of datasets. We can see that using the Chi square distance function is the best distance metric for *k*-NN over most of the datasets, which are contraceptive, Liver_disorders, and Stalog. On the other hand, *k*-NN by the Euclidean (and Minkowsky) distance function does not outperform *k*-NN by the other distance functions for these four datasets.

**Fig. 7:**
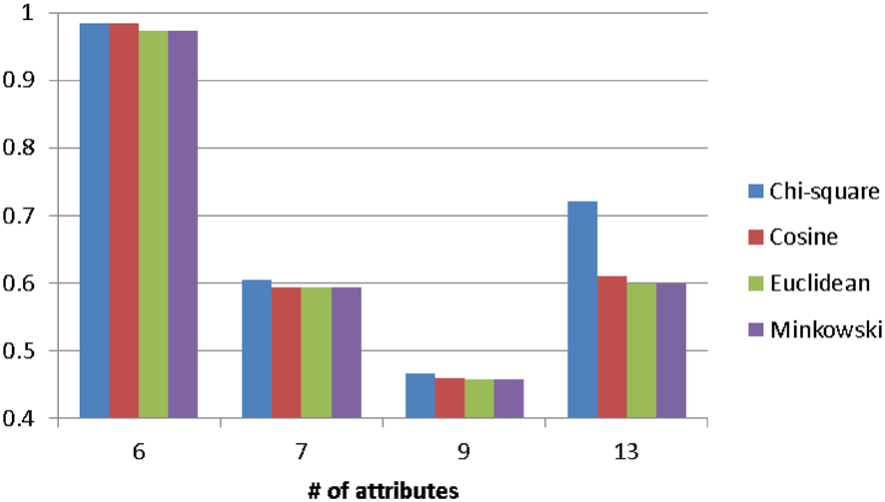
The average classification of *k*-NN over the attribute numbers of the four mixed types of datasets

Figure 7 shows the average classification accuracy of *k*-NN over the attribute numbers of the four mixed types of datasets. When the number of attributes increases, using the Chi square distance function is the better choice for *k*-NN.

#### Further comparisons

**Fig. 8:**
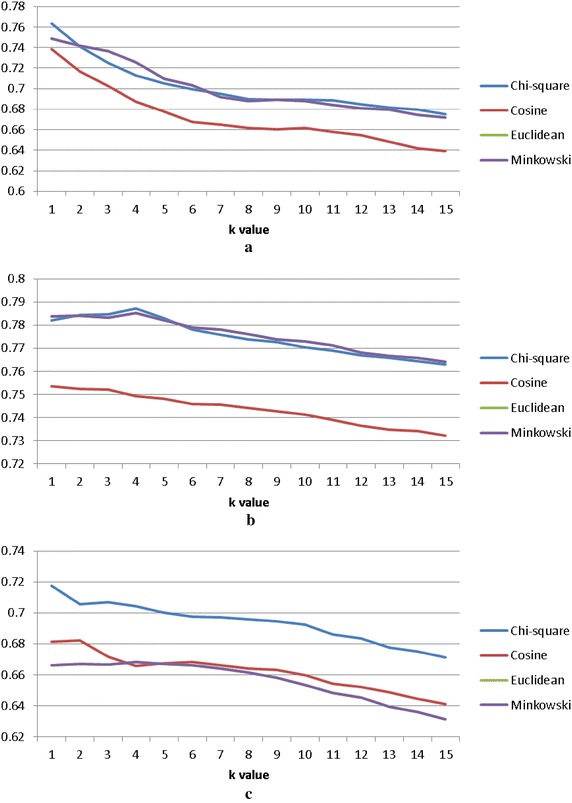
Average classification accuracy of *k*-NN. **a** Over categorical datasets, **b** over numerical of datasets and **c** over mixed types of datasets

Figure 8 shows the average classification accuracy of *k*-NN over categorical, numerical, and mixed types of datasets respectively. Overall speaking, we can observe that using the Chi square distance function is the best choice for the categorical, numerical, and mixed types of datasets whereas *k*-NN by the cosine and Euclidean (and Minkowsky) distance function perform the worst over the mixed type of datasets.

## Conclusions

In this paper, we hypothesize that since *k*-NN classification is based on measuring the distance between the test data and each of the training data, the chosen distance function can affect the classification accuracy. In addition, as different medical domain problem datasets usually contain different types of data, such as the categorical, numerical, and mixed types of data, these three types of data are considered in this paper.

By using four different distance functions, which are Euclidean, cosine, Chi square, and Minkowsky, our experimental results show that *k*-NN by the Chi square distance function can make the k-NN classifier perform the best over the three different types of datasets. On the other hand, using the Euclidean distance function performs reasonably well over the categorical and numerical datasets, but not for the mixed type of datasets.

## Footnotes

1 Minkowsiki distance is typically used with *r* being 1 or 2, where the former is sometimes known as the Manhattan distance and the latter is the Euclidean distance.

2 http://archive.ics.uci.edu/ml/.
